# A Retrospective Analysis of the Effect of Subretinal Hyper-Reflective Material and Other Morphological Features of Neovascular Age-Related Macular Degeneration on Visual Acuity Outcomes in Eyes Treated with Intravitreal Aflibercept over One Year

**DOI:** 10.3390/vision2010005

**Published:** 2018-01-31

**Authors:** King Fai Calvin Leung, Susan M. Downes, Victor Chong

**Affiliations:** 1 Department of Medicine, University of New South Wales, Sydney NSW 2052, Australia; 2 Oxford Eye Hospital, University of Oxford, Oxford OX1 2JD, UK

**Keywords:** neovascular age-related macular degeneration, subretinal hyper-reflective material, spectral-domain optical coherence tomography, biomarker, aflibercept

## Abstract

A retrospective study of 176 treatment-naïve eyes with neovascular age-related macular degeneration (nAMD) that had undergone intravitreal aflibercept treatment (2.0 mg, 7–8 times over one year) was performed to correlate the effect of aflibercept on the morphological features of nAMD—subretinal hyper-reflective material (SHRM), pigment epithelial detachment (PED), subretinal fluid (SRF), and intraretinal fluid (IRF)—with visual acuity at baseline and at one year. Spectral-Domain Optical Coherence Tomography (SD-OCT) images and best-corrected visual acuity (BCVA) at baseline and at one year were obtained. The relationship between visual acuity and the presence of morphological features at baseline and at one year was statistically analysed. The proportion of eyes with PED (*p* = 0.01), SRF (*p* ≤ 0.001), and IRF (*p* ≤ 0.001) reduced at one year. SHRM (*p* = 0.002) and IRF (*p* = 0.0001) were associated with poorer baseline BCVA. The presence of SRF at baseline was associated with better baseline BCVA (*p* = 0.004) and 5.3 letters of improvement of BCVA after one year of treatment (*p* = 0.0001). For each letter increase in BCVA at baseline, 0.25 fewer letters were gained in BCVA at one year. While aflibercept can improve morphological abnormalities in nAMD, this is not always accompanied by a corresponding improvement in visual acuity.

## 1. Introduction

The management of neovascular age-related macular degeneration (nAMD) was revolutionised by the introduction of intravitreal vascular endothelial growth factor (VEGF) inhibition [1]. The use of anti-VEGF agents has lead to major improvements in treatment and prognosis in nAMD [2]. However, there are concerns regarding the maintenance and consistency of efficacy of anti-VEGF drugs in different patients [3]. A tendency for the treated eye to develop resistance to anti-VEGF over time has been observed [4], as well as the unpredictability of disease reactivation following stabilisation [5]. Recurrent neurosensory disturbance with ensuing damage as seen in untreated nAMD invariably leads to irreversible loss of vision [6], and thus an improved understanding of factors that affect the interaction between treatments and nAMD progression may help to address these concerns and improve predictability of visual outcomes.

**Figure 1:**
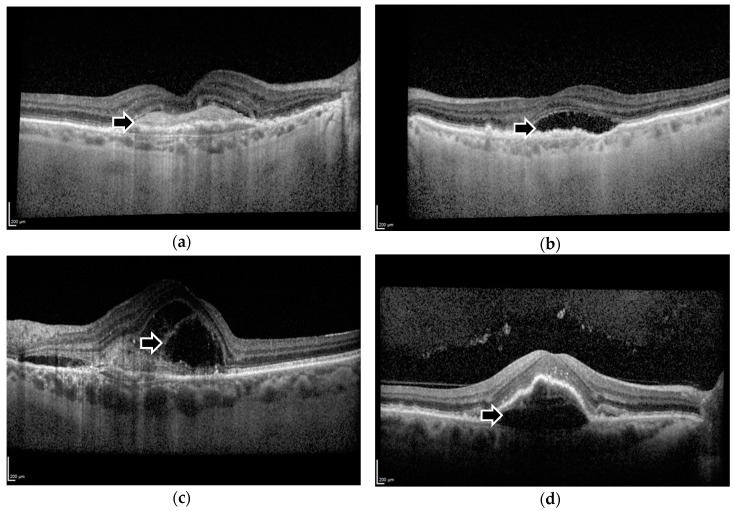
Spectral-Domain Optical Coherence Tomography images demonstrating (**a**) subretinal hyper-reflective material, (**b**) subretinal fluid, (**c**) intraretinal fluid, and (**d**) pigment epithelial detachment. Black arrows indicate the respective morphological features.

Morphological features such as intraretinal fluid (IRF), subretinal fluid (SRF), and pigment epithelial detachment (PED) have been proposed as potential biomarkers for monitoring the effect of intravitreal treatments on nAMD progression (Figure 1b–d). Aflibercept, a soluble decoy receptor fusion protein that inhibits VEGF [7], has been demonstrated in previous studies to enable resolution of these morphological features, and these improvements were correlated with gains in visual function [8,9].

Of increasing relevance as a biomarker is subretinal hyper-reflective material (SHRM), an anatomical feature seen on optical coherence tomography (OCT) imaging (Figure 1a). SHRM is hypothesised to be composed of a variable and dynamic combination of fluid, fibrin, blood, scar, and choroidal neovascularization (CNV) [10] (Figure 1a). It is located between the neurosensory retina and retinal pigment epithelium, and its presence is associated with reduced visual acuity and an increased occurrence of scarring [10]. SHRM has a clear role as a biomarker or prognostic factor in nAMD management, as the CATT study had previously found a decrease in the prevalence of SHRM in eyes treated with ranibizumab or bevacizumab [10]. This is significant, because a previous study of Time-Domain (TD) OCT images found that SHRM was present in over 70% of treatment-naïve eyes [11]. However, our recent study of a smaller patient cohort demonstrated that SHRM was only moderately common at baseline at 29.3% after analysis of images obtained via Spectral-Domain (SD) OCT [12]. SD-OCT has a greater resolution and thus is better at identifying exudative disease activity than TD-OCT [13]. Unfortunately, recent trials that have targeted SHRM with anti-platelet derived growth factor (PDGF) and anti-VEGF agents failed to demonstrate a significant improvement of best corrected visual acuity over or SHRM resolution when compared with anti-VEGF monotherapy [14,15,16,17,18].

Therefore, the impact and utility of the proposed biomarkers of nAMD on visual outcomes and the effect of anti-angiogenic therapy on these features are yet to be fully evaluated. Ultimately, the characterisation of these factors may well be important in personalised medicine in the context of nAMD treatment. Optimising functional outcomes, minimising disease recurrence, halting permanent retinal damage will lead to a significant reduction in the burden of care for consumers and providers [19,20].

To further explore the role of SHRM, IRF, SRF, and PED in its influence on nAMD treatment outcomes, we performed a retrospective analysis of the anti-exudative efficacy of aflibercept (Eylea, Regeneron, Tarrytown, NY, USA) on treatment-naïve eyes after one year of treatment with the recommended protocol [8,21]. Specifically, we investigated the impact of aflibercept on morphological features, correlating these with baseline visual acuity and change in visual acuity, with a focus on whether the presence of SHRM at baseline has an impact on treatment outcomes after one year of intravitreal aflibercept injections. 

## 2. Materials and Methods 

### 2.1. Study Design and Population

A retrospective analysis was performed to investigate the change in visual acuity and foveal morphology in treatment-naïve patients with nAMD after one year of intravitreal aflibercept treatment. Patients presenting consecutively between November 2013 and February 2015 to the Oxford Eye Hospital were included. Inclusion criteria included age older than 50 years, active and untreated CNV involving the foveal centre, VA between 20/40 and 20/200, and the completion of the recommended 12-month regimen of intravitreal aflibercept injections into the affected eye as in the pivotal clinical trial (VIEW program) [8]. This involved intravitreal injections of 2.0 mg of aflibercept monthly for the first three months, followed by fixed dosing every eight weeks, such that a completed regimen included 7–8 injections over 12 months [21]. Only one eye per patient was included in this study. In total, 176 eyes were included in this study.

This study was approved by the local ethical and research committee. 

### 2.2. Data Collection

The best-corrected visual acuity (BCVA) was assessed with logMAR charts at 4 m at baseline and prior to each injection by certified examiners at the Oxford Eye Hospital. In this study, BCVA data was obtained from electronic records. Data at baseline (before treatment) and at the study endpoint (one year after the start of treatment) were retrieved. LogMAR scores were converted to logMAR letters for this analysis.

**Figure 2:**
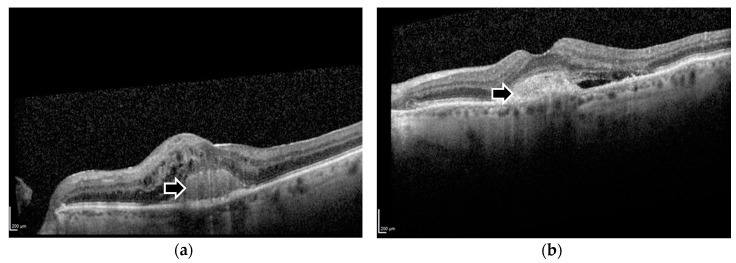
Spectral-Domain Optical Coherence Tomography images demonstrating foveal subretinal hyper-reflective material that is (**a**) distinct and (**b**) indistinct from the underlying retinal pigment epithelium. Black arrows indicate the respective morphological features.

Optical coherence tomography (OCT) images were obtained using the Spectralis SD-OCT device (Heidelberg, Germany) for clinical diagnosis and disease monitoring during the patient’s treatment. In this study, images taken at baseline and at one year were obtained from the image database at Oxford Eye Hospital. The evaluation of SHRM on OCT images was performed by 2 trained reviewers, and any indiscernible grades was determined by a senior reader (Victor Chong). For this analysis, eyes with SHRM were grouped together regardless of whether SHRM was distinct or indistinct from the retinal pigment epithelium complex (Figure 2). In addition to SHRM, each image was analysed by a trained observer at the Oxford Eye Hospital for the presence of any of the following: pigment-epithelial detachment (PED), subretinal fluid (SRF), and intraretinal (cystoid) fluid (IRF) within the foveal centre. In cases of ambiguity of the presence or absence of a morphological feature, a senior retinal specialist confirmed the final reading.

### 2.3. Statistical Analysis

Frequencies and percentages of each studied characteristic—sex of the patient, laterality, number of injections in one year, morphological features (SHRM, PED, SRF, IRF)—were determined within our sample population. A correlation analysis was performed to compare the relationship between the ages of each patient at first injection with either baseline BCVA or change in BCVA over one year to assess whether age was a potential confounding factor. Independent-samples *t*-tests were performed to identify other confounding predictors of BCVA at baseline and its change after one year of treatment, including laterality, sex, and number of injections. Paired student *t*-tests were performed to compare the presence of PED, SRF, and IRF at baseline and after one year of treatment with aflibercept. Independent-samples *t*-tests were performed to compare baseline BCVA with each morphological feature at baseline. The final BCVA was further divided into three groups—normal to near normal BCVA (greater than or equal to 76 letters or 20/32), visually impaired (66 to 74 letters, or between 20/40 and 20/63), and low vision (less than or equal to 65 letters or 20/80)—and Pearson’s Chi-square tests were performed to identify the association between baseline morphological features and the corresponding final BCVA group. Multiple regression analyses were performed to analyse the impact of baseline presence of SHRM, and of baseline and one-year changes in retinal morphological features—PED, SRF, and IRF—on the change in BCVA (in logMAR letters) at one year. The patient’s baseline BCVA was also included as an additional predictor in this model. To assist in this analysis, the effect of aflibercept on each morphological feature after one year of treatment was categorized into two groups: “improving” (morphological feature present at baseline but absent at one year) or “not improving” (morphological feature present or absent at baseline and one year, or absent at baseline and present at one year). Baseline morphological features were categorized as either “present” or “absent”. All statistical analyses were performed with the SPSS statistical package (IBM Corp, Armonk, NY, USA). Statistical significance was determined at *p* < 0.05.

## 3. Results

### 3.1. Baseline Characteristics

In total, 176 eyes with nAMD were included in this study, of which the left to right eye ratio was 1:1 and the male to female patient ratio was 1:1.7. Of these, 79 eyes (44.9%) had received seven injections after one year, while 97 eyes (55.1%) received eight injections over the same period of time. Baseline BCVA and change in BCVA after one year did not differ between groups for laterality, gender, and number of injections (Table 1). Out of the 176 eyes, 51 had SHRM (29.0%), while the remaining 125 did not have SHRM on SD-OCT (71.0%). The mean age was 81.3 and 80.5 years for patients with SHRM and without SHRM respectively (*p* = 0.489). The prevalence of SHRM did not differ between left and right eyes. There was no significant correlation between the age of the patient and baseline (*r* = −0.125, *p* = 0.097) or change in BCVA (*r* = −0.008, *p* = 0.920). The prevalence of other morphological features at baseline is described in the next section. See Table 1 for more information regarding frequencies.

### 3.2. Visual Acuity and Morphological Outcomes

The mean BCVA at baseline was 50.9 letters (or 20/100 + 1 in Snellen ratio) for eyes with SHRM and 58.7 letters (20/63 − 1) for those without SHRM (*p* = 0.002). At one year, the BCVA did not differ significantly between patients with SHRM and without SHRM (55.8 vs. 61.1 letters or 20/80 vs. 20/63 + 1 respectively, *p* = 0.064). There was therefore a change in BCVA of 5.0 and 2.4 letters after one year for patients with and without SHRM respectively. This change in BCVA was not significantly different between groups (*p* = 0.200).

At baseline, the number of eyes with PED was 58 (33.0%), 54 (30.7%) with SRF, and 73 (41.5%) with IRF. Following 7–8 intravitreal injections of aflibercept at one year, PED resolved in 23 eyes (39.7%), SRF in 48 eyes (88.9%), and IRF in 70 eyes (95.9%). There was a significant decrease in the prevalence of these morphological features after one year of intravitreal aflibercept (*p* = 0.01, *p* ≤ 0.001, *p* ≤ 0.001 respectively). Interestingly, the absence of SHRM at baseline correlated with better resolution of PED after aflibercept treatment within this study population (*p* = 0.013).

At baseline, BCVA did not differ between eyes with and without PED (56.6 vs. 56.16 letters or 20/80 + 2 vs. 20/80 + 1 respectively, *p* = 0.868). However, the presence of IRF was associated with poorer BCVA at baseline relative to the absence of this morphological feature (50.6 vs. 60.6 letters or 20/100 + 1 vs. 20/63 + 1 respectively, *p* = 0.0001). On the other hand, the presence of SRF at baseline was associated with a better baseline BCVA (61.2 vs. 54.3 letters or 20/63 + 1 vs. 20/80 − 1, *p* = 0.004). There was also a significant association between the one year visual function and whether there was SRF or IRF at baseline in corresponding eyes (*p* ≤ 0.001 and *p* = 0.011, respectively). In this latter analysis, final visual function at one year was defined by three groups—normal to near normal BCVA (greater than or equal to 76 letters or 20/32), visually impaired (66 to 74 letters, or between 20/40 and 20/63), and low vision (less than or equal to 65 letters or 20/80) (Table 2).

The effect of baseline morphological features on the extent of BCVA improvement after one year of intravitreal aflibercept injections into the affected eye was further explored using our multiple regression model. The presence of SRF at baseline was associated with a gain of 5.3 letters of BCVA after one year of aflibercept (*p* = 0.006). By contrast, for each letter increase in BCVA at baseline, there were 0.25 letters less gain in BCVA from baseline to one year (*p* < 0.001). Other predictors included in the model—presence of SHRM, PED, and IRF at baseline—did not demonstrate a significant correlation with BCVA improvement after one year (*p* > 0.05; R = 0.351, R^2^ = 0.123, adjusted R^2^ = 0.097, standard error of the estimate = 11.223) (Table 3). Multiple regression analyses of resolution in morphological biomarkers and baseline BCVA, and its effect on the change in BCVA after one year, demonstrated similar results, with only SRF improvement and baseline BCVA showing a significant correlation with change in BCVA, while resolution of PED and IRF had no significant correlation with visual function improvement (R = 0.334, R^2^ = 0.112, adjusted R^2^ = 0.86, standard error of the estimate = 11.295) (Table 4). The resolution of SRF at one year was associated with a gain of 4.9 letters of BCVA from baseline (*p* = 0.013). For each letter increase in BCVA at baseline, there were 0.25 letters less gain in BCVA from baseline to one year (*p* < 0.001). Age at first injection, sex, laterality of treated eye, and the number of injections administered after one year were excluded from the multiple regression model because these were found to be insignificant in initial statistical analyses.

## 4. Discussion

In this retrospective study, we found that after one year of the recommended regimen of intravitreal aflibercept injections [8,21], a resolution or a decrease in size of both SRF and IRF was observed in most treated eyes. While the therapeutic effect of aflibercept on PEDs was consistently lower, there nevertheless was an overall decrease in prevalence of PEDs, SRF, and IRF after one year of treatment. Previous literature has shown that treatment with aflibercept has been associated with a more rapid reduction in PED size in comparison to other anti-VEGF therapies. Our analyses further suggest that certain retinal morphological characteristics are associated with better or worse visual acuities at baseline [22]. For example, the presence of SRF was associated with better visual function in treatment-naïve eyes. However, the presence of IRF or SHRM at baseline was associated with poorer baseline visual acuities, and this is consistent with past findings [20,23]. 

In the analysis of the predictors of final visual outcomes, our data showed that baseline BCVA played an important role—the worse the initial visual function, the more the improvement in BCVA after one year of aflibercept therapy. This observed correlation may be partially explained by a “ceiling effect” of the degree of improvement possible in eyes with better baseline BCVA. Furthermore, the interesting relationship between SRF and BCVA became apparent again within our multiple regression analyses, showing that the presence of SRF at baseline and its resolution after one year were both associated with better final visual acuity scores. This apparently ‘protective’ aspect of SRF was also demonstrated in a past study by [24]. It has been postulated that the reason for better visual function in treatment-naïve eyes with SRF is that this represents an early stage of the pathophysiological course of nAMD. Fluid may initially reach the subretinal compartment due to the anatomical location of the proliferating choriocapillaris. IRF follows with disease progression, representing a stage that culminates with neurosensory functional loss, and IRF is also commonly seen in areas of fibrotic tissue. Early detection and treatment before the development of IRF may potentially improve visual function outcomes.

Since SHRM has recently emerged as a possible biomarker, we were interested in investigating whether there is a possible interaction between this morphological feature and its response to aflibercept treatment and overall response of the nAMD as measured by anatomical features and visual acuity. The CATT study previously found a decrease in the prevalence of SHRM in eyes treated with ranibizumab or bevacizumab [14]. Our study demonstrated that while the presence of SHRM is associated with poorer baseline BCVA, it was not significantly correlated with an improvement in visual acuity after one year of intravitreal aflibercept injections. Thus, aflibercept does not appear to confer a beneficial effect in nAMD eyes with SHRM. However, future studies of the interaction between aflibercept and SHRM should involve an examination of the effect of aflibercept on the prevalence and size of SHRM, and whether a change in SHRM size is correlated with visual acuity changes.

The limitations of this study are primarily associated with its retrospective design. Our results are limited by potential patient selection bias and a lack of control of both the method of measuring outcomes as well as the confounding factors relevant to our analyses. Nonetheless, it is a consecutive cohort of all the patients from our study population that were treated with the standard treatment protocol as published in the pivotal clinical trial (VIEW program) [8,21]. In this study, patients were excluded due to lack of completion of the treatment regimen or missing OCT and visual data, which may have introduced bias into the study. Patients that completed the treatment protocol may differ from those that did not follow the regimen in terms of health or socioeconomic status. In contrast to previous studies [20,24], our results did not demonstrate a significant effect of IRF on visual acuity outcomes, nor was there a significant association between morphological improvement and BCVA change, suggesting that our sample size may have been too small. Moreover, our use of a binary classification of each of the morphological features does not accurately describe the complex nature of retinal anatomy and the lesions found in nAMD. Future analyses should therefore also include the dimensions, composition, and timing of appearance of each retinal morphological feature.

In conclusion, this retrospective study demonstrated that aflibercept is efficacious in resolving the morphological abnormalities seen during active nAMD. The presence of SHRM and IRF at baseline was associated with poorer baseline BCVA, while eyes with SRF demonstrated better initial visual function. Baseline BCVA and SRF were respectively found to be negatively and positively correlated with change in BCVA after one year of intravitreal aflibercept injections, but similar effects from improvements of PED, IRF, and SHRM on BCVA were not observed. Further analyses of the precise effect of aflibercept on retinal morphology and visual acuity with a larger sample size and potential prospective studies looking at similar factors will help to assess the value of different morphological biomarkers and their relationship to current nAMD treatments, potentially introducing a new personalized approach to the management of this major cause of retinal blindness.

## Figures and Tables

**Table 1:** Characteristics of the eyes included in this study and their frequencies of occurrence.

Characteristics	Number of Eyes (Percentage)	Mean BCVA in Letters (Standard Error of the Mean), and Approximate Snellen Ratio		*p*-Value
**Gender**				
Male	66 (37.5)	Baseline BCVA	56.24 (1.917), 20/80 + 1	0.901 ^a^
		One year BCVA	61.52 (1.903), 20/63 + 2	0.212 ^a^
		Change in BCVA	5.27 (1.291)	0.054 ^a^
Female	110 (62.5)	Baseline BCVA	56.54 (1.394), 20/80 + 2	
		One year BCVA	58.41 (1.590), 20/80 + 3	
		Change in BCVA	1.87 (1.183)	
**Laterality**				
Left	88 (50.0)	Baseline BCVA	56.89 (1.537), 20/80 + 2	0.684
		One year BCVA	60.43 (1.795), 20/63	0.485
		Change in BCVA	3.55 (1.301)	0.656
Right	88 (50.0)	Baseline BCVA	55.97 (1.653), 20/80 + 1	
		One year BCVA	58.72 (1.674), 20/63 − 1	
		Change in BCVA	2.75 (1.222)	
**Number of Injections in One Year**				
7	79 (44.9)	Baseline BCVA	55.67 (1.657), 20/80 + 1	0.545
		One year BCVA	59.35 (1.891), 20/63 − 1	0.873
		Change in BCVA	3.68 (1.221)	0.583
8	97 (55.1)	Baseline BCVA	57.04 (1.539), 20/80 + 2	
		One year BCVA	59.75 (1.612), 20/63	
		Change in BCVA	2.71 (1.277)	
**SHRM**				
Present at baseline	51 (29.0)	Baseline BCVA	50.86 (2.128), 20/100 + 1	0.002
		One year BCVA	55.84 (2.425), 20/80 + 1	0.064
		Change in BCVA	4.98 (1.705)	0.200
Absent at baseline	125 (71.0)	Baseline BCVA	58.70 (1.277), 20/63 − 1	
		One year BCVA	61.10 (1.397), 20/63 + 1	
		Change in BCVA	2.40 (1.040)	
**PED—Baseline**				
Present	58 (33.0)	Baseline BCVA	56.16 (2.002), 20/80 + 1	0.868
		One year BCVA	60.29 (2.239), 20/63	0.689
		Change in BCVA	4.14 (1.833)	0.479
Absent	118 (67.0)	Baseline BCVA	56.56 (1.367), 20/80 + 2	
		One year BCVA	59.22 (1.465), 20/63 − 1	
		Change in BCVA	2.66 (0.978)	
**PED—One Year**		*One year BCVA*		0.978
Present	44 (25.0)	59.64 (2.638), 20/63		
Absent	132 (75.0)	59.55 (1.383), 20/63		
**PED—Resolution**		*Change in BCVA*		0.992
Improvement	23 (13.1)	3.17 (2.834)		
No improvement	153 (86.9)	3.14 (0.935)		
**SRF—Baseline**				
Present	54 (30.7)	Baseline BCVA	61.24 (1.847), 20/63 + 1	0.004
		One year BCVA	66.80 (2.065), 20/50 + 2	0.0001
		Change in BCVA	5.56 (1.312)	0.047
Absent	122 (69.3)	Baseline BCVA	54.30 (1.364), 20/80 − 1	
		One year BCVA	56.38 (1.425), 20/80 + 1	
		Change in BCVA	2.08 (1.136)	
**SRF—One year**		*One year BCVA*		0.394
Present	8 (4.5)	63.50 (4.396), 20/50 − 1		
Absent	168 (95.5)	59.39 (1.267), 20/63 − 1		
**SRF—Resolution**		*Change in BCVA*		0.073
Improvement	48 (27.3)	5.50 (1.411)		
No improvement	128 (72.7)	2.27 (1.097)		
**IRF—Baseline**				
Present	73 (41.5)	Baseline BCVA	50.55 (1.703), 20/100 + 1	0.0001
		One year BCVA	54.97 (1.917), 20/80	0.002
		Change in BCVA	4.42 (1.238)	0.215
Absent	103 (58.5)	Baseline BCVA	60.59 (1.363), 20/63 + 1	
		One year BCVA	62.83 (1.521), 20/63 + 3	
		Change in BCVA	2.24 (1.240)	
**IRF—One Year**		*One year BCVA*		0.318
Present	5 (2.8)	53.20 (5.687), 20/80 − 2		
Absent	171 (97.2)	59.76 (1.249), 20/63		
**IRF—Resolution**		*Change in BCVA*		0.205
Improvement	70 (39.8)	4.50 (1.288)		
No improvement	106 (60.2)	2.25 (1.206)		

^a^ male vs. female.

**Table 2:** Correlation between baseline morphological features and one year functional best corrected visual acuity.

	Total Eyes (*n* = 176)	SHRM	No SHRM	PED	No PED	SRF	No SRF	IRF	No IRF
**Normal ^a^**	43	11	32	11	32	25	18	15	28
**Impaired ^b^**	26	7	19	11	15	7	19	5	21
**Low ^c^**	107	33	74	36	71	22	85	53	54
***p*-Value ^d^**		*p* = 0.789		*p* = 0.348		*p* ≤ 0.001		*p* = 0.011	

^a^ BCVA greater than or equal to 75 letters; ^b^ BCVA between 66 to 74 letters, or between 20/40 and 20/63; ^c^ BCVA less than or equal to 65 letters or 20/80; ^d^ Using Pearson’s Chi-square test of independence.

**Table 3:** Multiple regression model to demonstrate the effect of visual acuity and morphological features at baseline on the degree of change in visual acuity after one year of intravitreal aflibercept injections.

**Model Summary**							
R		R^2^		Adjusted R^2^		Std. Error of the Estimate	
0.351 ^a^		0.123		0.097		11.223	
**ANOVA ^b^**							
	Sum of Squares		df	Mean Square	F		Sig.
Regression	3001.567		5	600.313	4.766		<0.001 ^a^
Residual	21,412.592		170	125.956			
Total	24,414.159		175				
**Coefficients ^b^**							
	Unstandardised Coefficients			Standardised Coefficients			
	B		Std. Error	Beta	t		Sig.
(Constant)	14.810		4.260		3.476		0.001
Baseline visual acuity	−0.249		0.064	−0.315	−3.888		<0.001
SHRM grading	0.707		1.961	0.027	0.360		0.719
PED at baseline	1.730		1.845	0.069	0.938		0.350
SRF at baseline	5.264		1.889	0.206	2.787		0.006
IRF at baseline	0.040		1.846	0.002	0.022		0.983

^a^ Predictors: (Constant), IRF at baseline, SHRM grading, SRF at baseline, PED at baseline, Baseline visual acuity; ^b^ Dependent variable: Change in visual acuity after one year.

**Table 4:** Multiple regression model to demonstrate the effect of baseline visual acuity and resolution of morphological features at one year on the degree of change in visual acuity after one year of intravitreal aflibercept injections.

**Model Summary**						
R		R^2^		Adjusted R^2^		Std. Error of the Estimate
0.334 ^a^		0.112		0.086		11.295
**ANOVA ^b^**						
	Sum of Squares		df	Mean Square	F	Sig.
Regression	2726.419		5	545.284	4.274	0.001 ^a^
Residual	21,687.741		170	127.575		
Total	24,414.159		175			
**Coefficients ^b^**						
	Unstandardised Coefficients			Standardised Coefficients		
	B		Std. Error	Beta	t	Sig.
(Constant)	15.899		4.122		3.857	<0.001
Baseline visual acuity	−0.251		0.064	−0.318	−3.912	<0.001
PED improvement or no improvement	0.890		2.549	0.025	0.349	0.727
SRF improvement or no improvement	4.945		1.980	0.187	2.497	0.013
IRF improvement or no improvement	−0.372		1.855	−0.015	−0.200	0.841
SHRM grading	0.399		1.957	0.015	0.204	0.839

^a^ Predictors: (Constant), SHRM grading, IRF improvement or no improvement, SRF improvement or no improvement, PED improvement or no improvement, Baseline visual acuity; ^b^ Dependent variable: Change in visual acuity after one year
